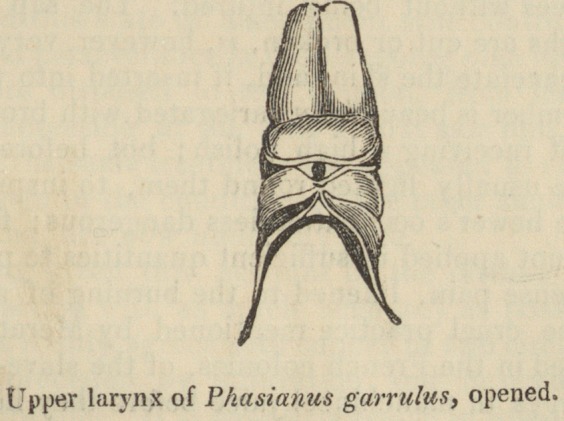# Collectanea: Miscellaneous

**Published:** 1834-04-01

**Authors:** 


### MISCELLANEOUS.

### LETTER FROM MR. MACILWAIN.

We have received the following letter from Mr. Macilwain:

*To the Editor of the Medical Quarterly Review.*

*9, Argyll Place; January* 15, 1834.

"Sir: My attention was yesterday directed by a pupil to your review of my "Observations on Porrigo," in which you transcribe a typographical error. As it materially affects the basis of your critique, and, what is of more consequence to me, renders the whole chapter unintelligible, I take the liberty of pointing it out to you. In the title of the chapter, for 'as they are *un*constitutionally,' read 'as they are constitutionally.' I inclose a list of the errata; and remain, sir, your obedient servant,

"G. Macilwain."

Now, though we readily admit that this correction is an improvement, we unhesitatingly deny that the original error formed the basis of our critique. It was based on a greater error,—on the whole of Mr. Macilwain's first chapter, as any one may see, by turning to our review. In quoting this chapter we omitted the note: we give it now.

"The consideration of the whole of the diseases of the skin strongly supports this view of the subject: as examples, I may refer to the Exanthemata, Lichen, Strophulus, Prurigo, which are in the order which I have placed their illustrations of these observations." (P. 7, *note.*)

The latter part of this sentence has no meaning; and, on refering to the list of errata with which the author has favoured us, we find, "Page 7, *note*, for 'I have placed there,' read 'I have placed them.'" This correction still leaves the sentence in a state more fit for Œdipus than Davus; and, moreover, the curious reader will observe, that there is an erratum in the erratum, "there" being substituted for "their." We think that authors deserve some little reproof even for typographical errors, when they make books unintelligible; but our objections to Mr. Macilwain's treatise were founded on our firm persuasion that it was essentially uninstructive. The plain truth is, that when a book intended for the public is examined by a professional man, the effect is like that which is produced by too near an approach to the gay illusions of the theatre: the spectator in the gallery thinks that he sees the enchanted bower of Armida, while the critic, on the third bench of the pit, can distinguish too clearly the coarse daubing and copper tinsel.

Our regret that Mr. Macilwain should have put forth a book like this, is increased by his being obviously capable of better things. The author of the paper on *Nævi*, in the last part of the Medico-Chirurgical Transactions, should not have written the "Clinical Observations."

### REGULATION OF MADHOUSES.

*To the Honourable the Commons, &c. the Petition of Caleb Crowther, Physician, practising in Wakefield, humbly sheweth,*

That your petitioner has, professionally, for many years attended the pauper lunatic asylum of this place, and that since he resigned the office of physician to that institution, he has visited a great number of madhouses and public hospitals, in different parts of the empire, with a view of comparing their respective merits and defects, and of forming a just estimate of the first principles necessary for the government of madhouses.

That your petitioner humbly prayeth your Honourable House to appoint a commission, during the present session of parliament, to examine, without exception, all the public and private asylums for the insane in the United Kingdom; and to compare their administration with that of some of the best regulated hospitals and infirmaries.

That the infirmaries at Glasgow, Liverpool, Manchester, Birmingham, and Leeds, in the opinion of your petitioner, afford admirable specimens of hospital discipline.

That the Asylum at Glasgow, and the Retreat belonging to the Society of Friends, at York, afford the most favourable examples of a public and private madhouse which your petitioner has witnessed.

That although great improvement has been made in the management of madhouses during the last twenty years, your petitioner has reason to believe that great abuses, great negligence, and great licentiousness, still exist in some of them.

That the first principles necessary for improving the management of madhouses, are daily visitation, scientific governors, the admission of patients in the first stage of the disease, and regular employment for the convalescent insane.

That the superiority of our infirmaries has, with great justice, been ascribed to the assiduity of their daily visitors; who minutely examine the cleanliness and ventilation of the house, the quantity and quality of the food, the conduct and behaviour of all the officers, servants, and patients in the institution. Whatever is found amiss is recorded in a book, submitted to the inspection of a weekly board of governors.

That this system of visitation has been adopted at the asylum at Glasgow, and at the Retreat at York.

That, without the aid of such a system, the visits of governors once in three months will ever remain ineffective.

That, in the opinion of your petitioner, there exist in every part of the country benevolent beings of both sexes, who would accept and faithfully execute such a trust gratuitously.

That the governors of madhouses ought either to understand the moral and medical management of the insane, or, like a sensible jury, they ought to be guided by those who do understand the subject.

That the great errors committed by the visiting justices, in medical matters, at the Penitentiary at Milbank, at Coldbath-Fields prison, at the asylum and prison at Wakefield, and at the asylum at Hanwell, prove how unfit magistrates are to govern such institutions, and indicate the necessity, in this country, of the appointment of a minister of health. That magistrates do not understand when the insane are judiciously treated, and will not submit to the appointment of visitors, because they say that it will create *imperium in imperio*, and derogate from their power.

Your petitioner, therefore, begs permission to suggest to your Honourable House the propriety of putting all madhouses, both public and private, under the management and entire direction of a medical board, consisting of twelve persons; nine or ten of that number to be physicians, and the remainder civil engineers. Three members of this board, two physicians and one civil engineer, to be required to visit four times a year, at irregular intervals, every asylum in the United Kingdom.

That correct reports of the history and treatment of every insane case be required to be transmitted monthly to this board, in London. That such reports would serve to stimulate the diligence, and to expose the neglect, of the medical attendants, and to improve and equalize the mode of treating the insane.

That, at each visit, the travelling physician ought to be required to examine every individual patient, and to ascertain the correctness of the reports transmitted to the board. Such a regulation would prevent the possibility of any insane patient being long unnecessarily confined. A report or summary of the practice in every asylum, both private and public, ought to be published annually by the medical board.

All the acts of the physicians and civil engineers ought to be done in public, except the visits of the physicians to the insane patients.

The business of the civil engineer will be, to examine whatever relates to the buildings, to the accounts, and to the domestic economy of the establishment.

That, at the first attack of the disease, the patient ought to be sent to an asylum, or privately put under proper restraint and suitable treatment, in order to prevent the disease from becoming incurable from injury done to the structure of the brain.

In the higher classes of society, as well as among the poor, the insane are frequently detained at home, in consequence of the fears, the ignorance, and the prejudices of their relations, and sometimes from the self-interest of the medical attendant, until the disease, from morbid organization, becomes incurable.

Your petitioner humbly submits to the consideration of parliament, whether or not a much larger fine ought to be imposed upon the rich, than what is now imposed, by the existing law, upon the overseers of the poor, who neglect to put their relations and friends under such restraint and medical treatment, as is essential for their safety and recovery. Your petitioner is, from ample experience, convinced that, after curing the bodily disease, incident upon the first attack of insanity, nothing contributes more towards removing the mental alienation, than employment suited to the circumstances and habits of the patient. The method of verifying this observation will be, to compare the number of patients cured in madhouses, placed under similar circumstances, where employment is extensively used, and where no recourse is had to it.

Your petitioner, therefore, most humbly prays that your Honourable House will adopt the measures herein suggested, or such other measures as may appear to your wisdom most efficacious, to secure to the insane judicious and humane treatment during confinement, and the most speedy restoration to their friends; and your petitioner will ever pray. CALEB CROWTHER, M.D.

*Med. Gazette.*

[We cordially agree with the greater part of this well-written petition; but on two points we must enter our dissent. We do not, in the first place, see the absolute necessity of the physician's companion being a civil engineer; a barrister, clergyman, private gentleman, or sharp half-pay major, might in many instances be advantageously substituted. In the next place, we must solemnly protest against any one being fined for not sending his mad, eccentric, or strangely-behaving relations to an asylum. Suppose a bachelor uncle, æt. sixty-three, to leave off his surtout, change his newspaper, learn to play at shorts, and avow a taste for French cookery; his sharp-set nephews and nieces have seen in print that these are indications of insanity, for that "as a general rule, any change from the usual habits of the individual should excite suspicion," (Liddell's translation of Esquirol on the Hallucinations of the Insane, p. 35. Note,) and would you add a fresh stimulus to their appetite in the shape of a fine ever suspended over their head, like the sword of Damocles? Are they not ready enough to lament over their uncle's eccentricities, and wonder what his canal shares may amount to, without the assistance of Will. v. cap. 188, § 1297?—*Ed. Med. Quart. Rev.*]

### GREAT OAKS.

The celebrated Cowthorpe oak, upon an estate near Wetherby, belonging to the Right Hon. Lady Stourton, measures, within three feet of the surface, sixteen yards in circumference, and close by the ground twenty-six yards. Its height is about eighty feet, and its principal limb extends sixteen yards from the boll. The Greendale oak, at a foot from the ground, is in circumference thirty-three feet ten inches. The Shire oak covers nearly 707 square yards; the branches stretching into three counties, York, Nottingham, and Derby. The Fairlop oak, in Essex, at a yard from the ground, is thirty-six feet in circumference. Damory's oak, in Dorsetshire, at the ground, was in circumference sixty-eight feet, and when decaying became hollow, forming a cavity capable of containing twenty men. An oak felled at Withy Park, Shropshire, in 1697, was nine feet in diameter without the bark. The Baddington oak, in the Vale of Gloucester, was fifty-four feet in circumference at the base; and Wallace's oak, in Torwood, in the county of Stirling, must have been at least eleven or twelve feet in diameter.—*Sir W. Jardine's Notes to White's Natural History of Selborne.*

This list might easily be extended. Professor Burnett mentions an oak in the hamlet of Kingsland, between London and Hackney, the cavity of which has been fitted-up as a chapel: the ingenious author of the "Outlines of Botany" was one of a congregation of nearly eighty persons assembled in it, and there was room to spare. At Creswell, in the county of Derby, there is an oak of vast size, and hollow. It happened some years since that two London workmen, engaged in decorating the duke of Portland's mansion at Welbeck, were reposing after their day's labour, and enjoying a can of beer within the genial recesses of this venerable tree. Unluckily their conversation took a botanical turn, and they talked of the tree in which they were sitting: one stoutly maintained it to be a beech, and the other as firmly asserted it to be an ash. To settle the dispute, one of them plucked a leaf, but this only aggravated the bitterness of the debate; for each appealed to the leaf as a proof of the stupid and almost malignant obstinacy with which his opponent persisted in denying the plainest matter of fact. Our fair informant, who witnessed the scene, and was at that time a very little girl, thought the disputants more than half-crazed, and ran home to tell her father of the surprising ignorance of a couple of Londoners.

### ON THE APPARENT DIRECTION OF EYES IN A PORTRAIT.

When we consider, says the author, the precision with which we commonly judge whether the eyes of another person are fixed upon ourselves, it is surprising that the grounds of such judgment are not distinctly known, and that most persons in attempting to explain the subject would overlook some of the circumstances by which they are generally guided. Though it may not be possible to demonstrate, by any decisive experiment, on the eyes of living persons what those circumstances are, we may find convincing arguments to prove their influence, if it can be shown in the case of portraits, that the same ready decision that we pronounce on the direction of the eyes is founded, in great measure, on the view presented to us of parts which have not been considered as assisting our judgment.

Dr. Wollaston then adverts to the influence of the form of the iris, as announcing the direction of the eye in portraits, and to that of the variable portion of the white shown when the eye is variously directed in living persons: he remarks, however, that even in real eyes we are not guided by this circumstance alone, but are unconsciously aided by the concurrent position of the face; and he illustrates this opinion by reference to a series of drawings annexed to the paper, and which show that the apparent position of the eyes is principally influenced by that of the adjacent parts of the face, especially those which are most prominent; and these considerations are not limited in their application merely to cases of lateral turn of the eyes and face. But the same principles also apply to instances of moderate inclination of the face upwards or downwards: for when the face is directed downwards, the eyes that look at us must be turned upwards, from the position of the face to which they belong; and if to eyes so drawn an upward cast of features be substituted for the former, the eyes immediately look above us. From these and other details given in the paper, the author concludes that the apparent direction of the eyes to or from the spectator, depends upon the balance of two circumstances combined in the same representation; namely, 1st, the general position of the face presented to the spectator; 2d, the turn of the eyes from that position; and thence proceeds to examine why, if the eyes of a portrait look at the spectator placed in front of the picture, they appear to follow him in every other direction. When two objects are seen on the ground at different distances from us in the same direction, one appears and must be represented exactly above the other, so that a vertical plane from the eye would pass through them; and since such a line will be seen upright, however far we remove to one side, it follows that the same objects still seem to be in a line with us exactly as in the front view, seeming as we move to turn from their first direction.

In portraits the permanence of direction, with reference to the spectator, and corresponding change of its apparent position in space when he moves to either side, depends upon the same principles. The nose drawn in front, with its central line upright, continues directed to the spectator, though viewed obliquely; or if the right side of the nose is represented, it must appear directed to the right of the spectator in all situations; so that eyes that turn in a due degree from that direction towards the spectator, so as to look at him when viewed in front, will continue to do so when viewed obliquely.—*Dr. Wollaston, in Abstract of Phil. Trans.*

### THE WEEPING WILLOW.

The weeping willow, (*S. Babylonica*,) which is the most ornamental species, has received its specific name from the supposition that it was upon such trees that the Israelites hanged their harps, when by the waters of Babylon they sat down and wept on the remembrance of Sion. But modern travellers affirm that it is a mountain-plant, and not an aquatic one. Pope's willow, at Twickenham, which was sacrilegiously cut down a few years ago, was the parent of many of those now growing in this country, as it was a favorite source; it is said to have been raised from a rod that with others formed the outer part of a package arrived from Spain, and which the poet planted, thinking it exhibited some signs of vitality.—*Burnett's Outlines of Botany.*

### BIRTHS IN PARIS DURING THE YEARS 1831 AND 1832.

In 1831 there were 29,530 births; in 1832 there were 26,823.

During fifteen years, *i. e.* from 1817 to 1831, there were born in France 7,490,951 boys, and 7,041,247 girls; the proportion of the former to the latter being nearly as seventeen to sixteen: that is to say, the number of males exceeded that of females by one-sixteenth. This, it will be observed, is at variance with the general opinion, which had fixed the proportion as being twenty-two to twenty-one. It is farther remarkable, however, that if we separate the natural children from those born in wedlock, the result brings us nearer to the old calculation: thus, in the fifteen years above alluded to, there were born in France, of illegitimate children, males 523, 436, females 501,115; being very nearly as twenty-three to twenty-two.—*Medical Gazette.*

### DEATHS IN PARIS IN 1831 AND 1832.

In 1831, the number of deaths was 25,996; in 1832, the number was 44,463: but of these, 18,602 died of cholera; and if this number be subtracted from the deaths in 1832, it gives 25,861 as the mortality of that year, or a diminution of 135, which diminution is accounted for by the smaller number of births during the latter year, in consequence of the extraordinary sickness which then prevailed. The general result clearly proving, what we have demonstrated on former occasions with regard to London and other towns in this country, that when they have been visited by cholera, the mortality has been the ordinary number of deaths + those caused by the epidemic.—*Ibid.*

### FAIRY-RINGS.

In this paper the author relates briefly some observations which he formerly made on the progressive changes of these rings, which appear to him to lead to a satisfactory explanation of their origin.

In the first place, he observed that some species of fungi were always to be found at the exterior margin of the dark ring of grass if examined at the proper season. This position of the fungi led him to conjecture that progressive increase from a central point was the probable mode of formation of the ring: and he thought it likely that the soil which had once contributed to the support of fungi, might be so exhausted as to be rendered incapable of producing a second crop. The defect of nutriment on one side would occasion the new roots to extend themselves solely in the opposite direction, and would cause the circle of fungi continually to proceed, by annual enlargement, from the centre outwards. The luxuriance of the grass follows as a natural consequence, as the soil of an interior circle is enriched by the decayed roots of fungi of the succeeding year's growth. Such a progressive enlargement, he remarks, had already been observed by Dr. Hutton on the hill of Arthur's Seat near Edinburgh; but Dr. Hutton had not attended to the production of fungi.

Dr. Withering, on the contrary, remarked the connexion of the rings with fungi, but had not noticed their progressive enlargement.

During the growth of fungi, the author observes, they so entirely absorb all nutriment from the soil beneath, that the herbage is often for a while destroyed, and a ring appears bare of grass, surrounding the dark ring; but after the fungi have ceased to appear, the soil where they had grown becomes darker, and the grass soon vegetates again with peculiar vigour.

For the purpose of observing the progress of various circles, he marked them by incisions for three or four years in succession, and found their annual increase to vary from eight inches to as much as two feet, according to the species of fungus to which they are owing; for he has observed as many as five species that have this mode of growth; *Agaricus campestris*, *Ag. orcades*, *Ag. procerus*, *Ag. terreus*, and the *Lycoperdon bovista.*

The author has had many opportunities of remarking, that when two circles interfere with each other's progress, they do not cross each other, but are invariably obliterated between the points of contact. The exhaustion occasioned by each obstructs the progress of the other, and both are starved; a circumstance which he considers as a strong confirmation of his hypothesis.

He has further remarked, in one instance, that different species of fungi appear to require the same nutriment; for, in a case of interference of a circle of mushrooms with another of puff-balls, the circles were, as in other cases, both obliterated between the points of union.

With the hope of ascertaining in what length of time a soil might recover the power of producing a fresh crop of fungi, a groove was cut along the diameter of a mushroom-ring, and a quantity of the spawn taken from its circumference was inserted along it; but the experiment unfortunately failed altogether, and the author had no opportunity of repeating the experiment.—*Dr. Wollaston, in Abstract of Phil. Trans.*

### ON THE STRUCTURE OF THE COVERING OF THE CORNEA.

(In a Letter from W. C. Wallace, Esq., New York, to W. Mackenzie, M.D., Glasgow.)

*Hudson-street, New York; 22d November,* 1833.

My dear Sir, * * * *

It is stated that the conjunctiva lines the eyelids, and is reflected over the eye-ball; and that it is a membrane between the mucous and the cutaneous structure, as it partakes of the diseases of both.

When the eye of an ox is immersed in hot water or vinegar, the anterior membrane coagulates, and may be separated from the cornea, and from that portion of the conjunctiva which it covers. The conjunctiva does not coagulate, neither can it be traced to the cornea, but seems inserted into the sclerotica. When the eye is macerated, and the conjunctiva dissected from the eye-ball, the conjunctiva may be cut through at its attachment, and as the anterior membrane overlaps it, there may be the appearance of a continuity of structure; but if the separation be commenced on the cornea, and be carried to the conjunctiva, the corneal covering will be found to overlap it for a short space, and to be a distinct membrane, as it can be completely separated from it. It may be compared to a small watch-glass, very thin at the edges, a little larger than the cornea, and placed over it and the contiguous conjunctiva. This is not a mucous membrane. It resembles the cuticle, in being composed of albumen, and in being easily regenerated when abraded.

Were the anterior membrane a continuation of the conjunctiva, the chemosis in severe catarrhal ophthalmia would not stop at the edge of the cornea, but would proceed over its surface. Were it a mucous membrane, the mucus secreted would impede vision. I do not recollect having seen pustules on any part of the eye that were not covered by this membrane.

I am your obliged friend,

W. C. Wallace.

—*Med. Gazette.*

TEST FOR HYDROCYANIC OR PRUSSIC ACID, AND METHOD OF APPRECIATING THE QUANTITY.

We are informed by Mr. John T. Barry that the nitrate of silver, in common with other salts of that metal, is so extremely delicate a test of the presence of hydrocyanic acid, that its detection is not difficult in a drop of water containing far less than the ten thousandth part of a grain of that poisonous agent. For instance, if one minim of the dilute medicinal solution be mixed with a pint of water, its presence may be demonstrated in a single drop of the mixture. But what is of more consequence is, that although the mixture be contaminated with various organic substances, such as those contained in articles of diet, milk, coffee, tea, porter, wine and soups, so far as is yet known, the test retains its sensibility unimpaired. Mr. Barry, however, thinks that this extreme sensibility, while it renders the evidence of the silver test conclusive as to the absence of prussic acid, will be of more limited service in establishing its presence, for, without adverting to the possibility of other volatile substances being hereafter discovered to have a similar effect on solution of silver, it is to be borne in mind that this re-agent indicates the existence of prussic acid in some esculent substances where it had previously been found, as well as in some new ones. Upon this branch of the subject medical jurists will probably think it right to collect information.

The application of the solution of silver is simple. The suspected fluid is to be acidulated by the addition of acetic acid, but so as to redden litmus paper in only the slightest degree. If excess of acid be already present, it is to be not quite neutralized by carbonate of soda. These precautions are adopted to retard the interference of ammonia or muriatic acid. Two or three drops quite cold are then put into a watch glass, and immediately covered by a plate of glass, whose under-surface, to the breadth of a pea, is moistened with solution of nitrate of silver, formed by dissolving one grain lunar caustic in one hundred grains distilled water.

If the inverted drop of silver solution retain its transparency unaltered, the absence of prussic acid is established; for, had it been present, the silver solution would in a few moments have become clouded by the formation of a white precipitate, an effect which, indeed, is almost instantaneous when the prussic acid is not excessively diluted. If, on the other hand, the precipitate appear, the conclusion must not be drawn that it is cyanuret of silver, until identified as such by two properties: first, its speedy re-solubility, as denoted by the clouded drop becoming again clear, when placed over a vessel of caustic ammonia, in which respect it differs from the silver compounds of iodine and bromine: and secondly, in retaining unchanged its pure white colour after exposure a few minutes to the sun's rays, or for a longer time, to daylight. As this property essentially distinguishes it from the compound of silver with chlorine, it is important to establish it by a separate experiment upon a somewhat larger portion of the precipitate, which should be obtained, by candlelight, by successively placing the inverted drop of nitrate of silver over renewed portions of the liquid in a saucer: as soon as the precipitate separates into distinct curd-like particles, it is ready for exposure to the solar rays.

Another property which distinguishes the cyanide (or cyanuret) of silver from the chloride, is, that upon being ignited in an open short glass tube, the cyanogen burns with a flame of the usual colour, leaving the metal pure, if sufficiently heated, a quality the more valuable as it furnishes an index to the proportion of prussic acid it represents, which upon ordinary occasions may be estimated as equal to one fourth the weight of residual silver.

When, acting upon this principle, it is desirable to ascertain the entire quantity of prussic acid, it is to be obtained by slowly distilling over, in nearly filled close vessels, about an eighth of the acidulated mixture under examination; rectifying it; re-acidulating by acetic acid; precipitating by slight excess of nitrate silver; washing with distilled water, only so long as the washings affect litmus paper; drying at 212°; weighing: and lastly, igniting and re-weighing.—*The London and Edinburgh Philosophical Magazine.*

### SPARK PRODUCED DURING THE FREEZING OF WATER BY ÆTHER

M. Julia Fontenelle states that M. Pontus, professor at the Royal College of Cahors, has communicated to him the following observation. It is well known to chemists that if a phial, terminated by a small tube one to two centimetres long, be filled with water as well as the tube, and surrounded with cotton moistened with æther, the water freezes during the evaporation of the æther under the receiver of the air-pump. On repeating this experiment, M. Pontus remarked, that some moments before the congelation occurs, a spark, visible in daylight, escapes from the small tube which terminates the phial. This phænomenon is so generally true, that every time that he perceived the spark, he concluded the congelation was about to take place; and, on the contrary, when he did not see it, he presumed that the congelation was not near. M. Pontus was never disappointed in his conclusions. M. Fontenelle states that he also has seen the spark, and that M. Becquerel had remarked to him a similar effect at the moment of the formation of crystals in solutions.—*Ibid.*

### HORSE SHOES.

The earliest nailed shoe of which there is any certain record was found at Tournay, in Flanders, buried along with him in the coffin of Childeric, king of France, who died in the year 481: a particular account of the opening of this tomb is given by Chifletius; and Montfaucon, in his Antiquities, states, that this shoe was with nail-holes in it, and that it fell to pieces on being handled. He has figured it, and from its size, one might readily suppose that it belonged to some favourite mule. So small a piece remained however of this shoe that the greatest part of the figure is supplied by the draftsman, and the holes are by no means decisive of its being a shoe of the nailed kind; for we may remark that the iron applied for the preservation of their socks or *hippopodes*, must have been also perforated in order to fix them on to the leathern soles, so that considerable uncertainty remains respecting this point; and the period also is only a century later than Vegetius.—*Bracy Clark on the Usages of the Ancients respecting the Shoeing of the Horse.*

### A SURGICAL ANECDOTE.

A young surgeon, with all those natural advantages of mind and manners which qualify a man to succeed in society, was appointed to a Dispensary which had lately been established in his native county. Surrounded by partial relatives and friends, and possessing qualities which entitled him to their esteem, it is not surprising that he soon acquired a very considerable reputation, and in a very few years became exclusively possessed of the general practice of a rich and populous neighbourhood. And here I may observe, that I can scarcely conceive a situation more enviable than that of a young and successful medical practitioner in the country. He must indeed be deficient in morals, manners, or education, if he is not the most popular man in the neighbourhood. Every moment of his time is either agreeably or usefully employed; respected and beloved by the poor, to whom he never appears but in the character of a benefactor, he passes through the country (whatever be its state of tumult and insubordination,) by night or by day like a being of a superior order; let destruction fall where it may, his property and person at least are held sacred. His occupation gives healthful exercise, not unmixed with pleasure; and if, in the course of his extensive rides through the country, he should chance to fall in with the hounds, why who is so welcome as the doctor? Health brings with it cheerfulness, and cheerfulness is the parent of kindness. Add to all this independence, with that most delightful of all feelings, which Mr. Edgeworth calls "the sense of success," and I think you have as many of the requisites of happiness as can well fall to the lot of humanity.

Well, our young friend possessed all these advantages, and he had besides the advantage of being received as a friend as well as a physician in the house of the lord of the manor, one of the most amiable and distinguished men in the country. His life passed on in this way for four or five years, every day adding to his reputation and happiness, until one fatal night, when he was called in haste to visit his patron and friend, who was suffering from a retention of urine. Having tried the usual means of relief in vain, he attempted to pass the catheter, but after two hours, spent in painful and ineffectual efforts, he is obliged to call for further assistance. Allow me here to explain the nature of the difficulty which he had to encounter, but was unable to overcome. Retention of urine in old persons is, nine times out of ten, caused by an enlargement of the middle lobe or portion of the prostate gland, which pushes up, in this way, into the cavity of the bladder, and pressing against the internal orifice of the urethra, prevents the escape of the urine. If the catheter be pressed backwards, or even upwards, its point bears against the projecting portion of the gland, and cannot, without perforating it, reach the cavity of the bladder. (Here Mr. C. exhibited a section of the bladder and urethra, with enlargement of the middle lobe of the prostate gland.) The point of the catheter was seen pressing against the projecting lobe, which prevented the instrument from passing into the bladder.

This difficulty gave time for the arrival of a young surgeon, who had been induced, by the extraordinary success of our friend, to establish himself in the same village; for observe, that there is not a village in Ireland, however small or remote, in which you will not find a competitor. "I fear," said the surgeon who had charge of the case, "that this is a serious affair. I apprehend we shall be obliged to puncture the bladder, but try what you can do with the catheter." The young man, having ascertained the real state of the case by examining the prostate through the rectum, drew from his pocket a long and deeply curved catheter of the middle size, and having passed it up to the obstruction, depressed his hand, while at the same time he withdrew about an inch of the stilette, and the instrument instantly slipped over the obstruction into the bladder. In a few moments the patient was out of pain and out of danger. Need I describe the different feelings with which the two young aspirants for public favour turned towards their respective homes. The one, loaded with the praises and benedictions of a grateful family, springs upon his horse, which scarcely seems to touch the ground until he reached his home, where, in the bosom of his anxious family, he recounts every circumstance of his success, and indulges in bright anticipations of future fame and independence. The other, passing unheeded (it may be for the last time,) through the silent hall, which lately rung with his welcome, returns with heavy steps to his cheerless home, ruminating as he goes with bitter but unavailing regret, on opportunities neglected and ruined hopes.—*Dr. Crampton's Lecture, in Med. and Surg. Journal.*

### ON SOUNDS INAUDIBLE BY CERTAIN EARS.

In this communication the author describes a peculiar insensibility to certain sounds in the ears of persons not otherwise deaf, which he was led to observe by trying different modes of lessening the sense of hearing in himself; when he found, that by closing the nose and mouth, and expanding the chest, the membrana tympani, thrown into a state of tension by external pressure, made the ear insensible to grave tones, without affecting the perception of sharper sounds. In this case the ear was insensible to all sounds below F marked by the bass cliff.

In the natural healthy state of the ear, there seems to be no limit to the power of discerning low sounds: but if we attend to the opposite extremity of the scale of audible sounds, and with a series of pipes, exceeding each other in sharpness, examine their effects successively upon the ears of different persons, we shall find considerable difference in their powers of hearing them, and see reason to infer that human hearing is more confined than has been supposed. Dr. Wollaston's attention was called to this circumstance by finding a person insensible to the sound of a small organ-pipe, which, with respect to acuteness, was far within the limits of his own hearing. By subsequent examination, this person's hearing was found to terminate at a note four octaves above the middle E of the pianoforte. Other cases of the insensibility of the ear of certain persons to high sounds are next adverted to: such as to the chirping of the grasshopper, the cricket, the sparrow, and the bat; the latter being about five octaves above the middle E of the piano. The limit of the author's own sense of hearing is at about six octaves above the middle E; and, from numerous trials, he is induced to think that, at the limit of hearing, the interval of a single note between two sounds may be sufficient to render the higher note inaudible, although the lower one is heard distinctly.

The range of human hearing includes more than nine octaves, the whole of which are distinct to most ears, though the vibrations of a note at the higher extreme are 600 or 700 times more frequent than those which constitute the gravest audible sound; and as vibrations incomparably more frequent may exist, we may imagine, says the author, that animals like the Grylli, whose powers appear to commence nearly where ours terminate, may hear still sharper sounds, which we do not know to exist; and that there may be insects hearing nothing in common with us, but endued with a power of exciting, and a sense that perceives the same vibrations which constitute our ordinary sounds, but so remote that the animal who perceives them may be said to possess another sense, agreeing with our own, solely in the medium by which it is excited, and possibly wholly unaffected by those slower vibrations of which we are sensible.—*Dr. Wollaston, in Abstract of Phil. Trans.*

### THE TAXODIUM.

The Taxodium is a native of America, growing abundantly in the southern parts of the United States, and likewise in Mexico. In the gardens of Chapultepec is one called the Cypress of Montezuma, which was in full vigour when that prince was on the throne, in the year 1520. It is now forty-one feet in girth, and apparently only in its prime. But another, far more remark-worthy, is described by Exter, as standing in the burial-ground of *Santa Maria de Tesla*, which the inhabitants of Oaxaca call *Sabino*. There are several noble trees in the same place; the largest, however, is the vegetable wonder, measuring 117 feet ten inches (French) in circumference, thirty-seven feet and a half in diameter, and about one hundred feet in height. This patriarch of the woods was mentioned by Cortez, who encamped his little army beneath its shade; and it is regarded with reverence almost approaching to religious veneration by the native Mexicaus. The height at which the admeasurements were taken is not mentioned; but, supposing them taken on the ground-level, there are several Taxodia mentioned by Michaux, as growing in the Floridas and Louisiana, which would nearly equal the great tree of *Oaxaca:* for he says they gave forty feet in girth above a conical base three or four times as large as the columnar trunk. The mean age of the Taxodia has been calculated to be from 4000 to 6000 years; and if such computations be correct, which however is more than doubtful, the great tree of *Oaxaca* may be coeval with the creation. Or, as the poet says,

> "Its cold and lengthened tracts of shade
> Rose on the day when sun and stars were made."

—*Burnett's Outlines of Botany*, No. 14.

### ON THE ORGANS OF THE VOICE IN BIRDS.

There is a great difference in the construction of the larynx in those animals which have an epiglottis and in those which have none. In the latter we do not find any thing resembling the cricoid and thyroid cartilages of the larynx of mammiferæ. In the amphibia, the glottis is situated in a round fleshy mass *(bourrelet)*, formed by the interweaving of nearly circular fibres: in birds it is cartilaginous, almost osseous, and tolerably uniform in its external form. In the genera *Pelecanus*, *Phasianus*, *Ardea*, and *Phœnicopterus*, the larynx has a triangular shape, the apex of the triangle being directed forwards, and its base or posterior part being fringed with little pointed, white, cartilaginous teeth. These tubercles, which border the glottis, are very broad and short in *Phasianus paraka* of the Orinoco; they form a white band in the *Ardea cocoes;* they cover, almost entirely, the glottis of the *Palamedea bispinosa*, and that also of the *Aras*, a family of the parrot tribe; but they are entirely wanting in some aquatic birds, as in the alkatras of the South Sea. The six designs in which I have represented the larynx of birds, present all these varieties of construction. The glottis is supported at its base by an osseous, broad, flat cartilage, sometimes crenated anteriorly, as in the Guyana pheasant, but pointed and elongated in the pelicans and *Phœnicopterus.*

This cartilage, to which I shall give the name of base (*socle*) has

*a* *b* *c*

*a*, "Socle" of *Pelecanus alcatras.* *b*, ditto of *Pelecanus olivaceus.* *c*, ditto of *Phasianus garrulus.*

not hitherto been well described. It has, on its upper surface, a membrane which divides it into two parts, and which proceeds from it at right angles. This membrane is triangular, and resembles the index of a sun-dial. In the living animal, it is generally visible at the centre of the opening of the glottis: it there forms a partition, the presence of which must contribute a great deal to modify sounds and render them more acute: it divides, so to speak, into two currents, the air which is driven by the inferior larynx towards the glottis. I was surprised to find that it was wanting in the glottis of the *Palamedea bispinosa.* This flat cartilage, thus furnished with a triangular membrane, is immediately attached to the rings of the trachea: it may be considered as a half-ring of a singular construction.

Besides this base, the glottis of birds is supported by four little bones, which are connected, two by two, in the form of compasses, the anterior pair of which is embraced by the posterior. I do not

*a* *b* *b*

*a*, Larynx of *Ardea cocoes* opened. *b*, Bonelets, or compass bones.

hesitate to call these bonelets; for, in tropical birds their substance is too dense for them to be ranked among the cartilages. They are triangular, diminish towards the extremities, and are joined in pairs by condyles. I have carefully copied those of the great heron with black neck and white floating comb, of the river Magdalena, and which constitutes an intermediate species between the *Ardea cocoes* and the *Ardea johannæ.* This handsome bird is four feet four inches high, when he stands with his neck elongated, and his greenish bill raised. The two anterior bonelets, or the branches of the anterior compass, (if I may be allowed the expression,) are immoveable, while the posterior compass, which is placed over and embraces the other, extends its branches by means of two fleshy muscles which are placed outside the bony glottis. These posterior bonelets might be likened to the arytenoid cartilages, while the anterior by their position might correspond with the thyroid cartilage of mammiferæ; but, in classes of animals whose entire organisation is so different, these comparisons, most frequently, only serve to lead to very inaccurate ideas.

It follows, from this construction of the superior larynx, that the rima or opening of the glottis, confined by the anterior bonelets, can scarcely ever become either widened or more contracted; and that it is by the action of the posterior branches, which are placed at the outer border of the glottis, that the internal capacity of this organ becomes changed. The voice is always projected through the same opening, but the rapidity with which this is effected depends as much upon the impulse it has received in the inferior larynx, as upon the approximation of the bonelets of the glottis.

But the modification of the sounds does not depend solely upon the extremity of the trachea, nor upon the form of the two larynxes. The whole trachea in some of the tropical birds, presents very extraordinary appearances. In the same species, the trachea in the male is sometimes twice the length of that in the female. Linnæus has already announced this in the description of the *Phasianus parraka.* We also meet with this difference in several swimming and wading birds of Europe, in storks, cranes, and herons; and M. Daubenton has observed it in the fine genus *Crax*, the *Koco* of the tropics, which has often served us for food in the woods. There is an extraordinary prolongation and sinuosity of the trachea in a new species of pheasant, which I shall describe under the name of *Phasianus garrulus.* This pheasant, which must not be confounded with the *Phasianus motmot,* is very common north of the equator, in the river Magdalena, in the province of Caracas, and in New Andalusia. Flocks of from sixty to eighty perch themselves upon the dead branches of contiguous trees, and fill the air with their piercing cries of *catacras? catacras?*

I found the length of the trachea in the male of this species, to be, from the superior larynx to the bronchi, fifteen inches seven lines, while the female's was but five inches four lines. That in the male first descends among the teguments under the sternum as far as the legs; it is then folded back (in nearly the same manner as M. Bonpland found in the bronchi of the crocodile), forms a considerable sinuosity in reascending, and then enters the lungs. The trachea of the female, which is shorter in the proportion of five to two, does not form this sinuosity, but enters, without folding itself, at once into the bronchi. Here then we have a bird in which the air forming his voice passes between the legs before arriving at the glottis. The Indians observe that the cry of the female of this species is much less shrill than that of the male, whose trachea presents so singular a construction. M. Cuvier has communicated to me the important observation, that, in the common pheasant (*Phasianus colchicus*) the trachea does not, in either sex, present this sinuosity, and that, in the *Cygnus canorus*, the female presents the elongation of this organ. It is remarkable that such differences should exist in species nearly resembling it.

In passing from Santa-Fé de Bogota to Quito, during the rainy season, in 1801, we found the banks of the Canca covered with the *Palamedea bispinosa*, the kamichi of Buffon, which approximates in size to the condor of the Andes. It is called by the inhabitants the Uitre de Sienega. It walks about very gravely in marshy places, uttering constantly a uniform cry, somewhat resembling the sound produced by a boy's whistle *(soufflet)*.

Staying some days at Buga, I had an opportunity of obtaining one of these birds, and of examining its larynx. I saw with surprise that it was rather its trachea than its inferior larynx which enabled it to produce such extraordinary sounds. This trachea diminishes in diameter from the bony glottis to the inferior larynx: it contracts in the proportion of two to three; but, before arriving at the inferior larynx, it suddenly becomes much wider; it becomes

Enlargement of the trachea in *Palamedea bispinosa*, with the insertion of the two muscles.

nearly five-sixths larger than the glottis itself, and this enlargement is nearly fourteen lines long. Lower down, the trachea contracts again and more than before; for, in entering the inferior larynx, it has not one fourth of its original diameter. I sketched on the spot this peculiar conformation of the *Palamedea bispinosa*, of which I

have scarcely met with any analogy in other birds which I have dissected. M. Cuvier, however, has seen two other examples of these sudden enlargements, in the *Anas clangula*, and in *Anas fusca;* but in these cases the swelling was of a spherical form, with a nearly circular disc, and different, in that respect, from that of the *Palamedea bispinosa*. In this two bundles of very long and slender muscles are attached to the enlarged part of the trachea; these draw it downwards, so that the larger rings compress those of the narrower part; a mechanism resembling that of some musical instruments, and which, without doubt, contributes to the production of the monotonous cries and cadences of this bird.

I shall not dwell long on the structure of the inferior larynx of birds. It would be difficult to add anything to a subject which M. Cuvier has treated of in a particular memoir. I have drawn, in the greatest possible detail, the inferior larynxes of the *Psittacus ararauna*, and of a new species of pelican of New Guinea, which I shall describe under the name of *Pelecanus olivaceus*. It has the habit of a *Plotus;* but its bill and its intermediate claw being serrated, indicate that it belongs to the genus *Pelecanus*. In one figure the various muscles are delineated, with which nature has furnished this inferior larynx, which is really an instrument of music. The other figure represents the sacs or valves in this bird, which are of an extraordinary size, being more than three lines deep and two broad; but in some tropical animals which have a very strong voice, this seems to depend on the structure of the superior larynx, rather than on that of the inferior. This is the case with *Phasianus garrulus*, in which I have represented the bonelets and

Upper larynx of *Phasianus garrulus*, opened.

the arytenoid cartilages bent forward, in order to display the internal structure of the glottis. I did not find sacs in the inferior larynx of this bird, but simply a bulging out and broadening of the last rings. The base of its inferior larynx is sustained by a cartilage, which I have not met with in any other animal of this class; it is a round, membranous, crenulated plate, upon which rises a small compressed bone. The want of sacs in the lower end of the larynx, in the *Phasianus garrulus*, is compensated by the mechanism of the superior larynx. Above the opening of the trachea there is a rima leading to two membranous pouches. In blowing through the bronchi into the trachea, these pouches are seen to swell. Valves are also wanting in the inferior larynx of the *Pelecanus fuscus*, but there are true sacs in the superior. In the glottis of the *Palamedea bispinosa*, whose trachea presents the extraordinary enlargement I have just described, there are folds having some analogy with the sacs which in man form the ligaments which go from the arytenoid cartilages to the thyroid. These folds seem to modify the voice of this bird; for in the inferior larynx, which I opened and have carefully drawn, there is nothing which can perform their functions. In the same class of animals, it is sometimes the glottis, sometimes the form of the trachea, and sometimes the inferior larynx, which modifies or gives character to the voice.—*Field Naturalist's Magazine*.

### THE HIPPOMANEÆ.

The Hippomane of the Greeks was an Arcadian plant, said to have the power of making horses mad. It was not improbably a species of *Euphorbia*, the acrid juices of which, flowing into the wounds inflicted by the thorns, would render most beasts outrageous, and therefore must not be mistaken for the poisonous distillation from raging mares, as described by Virgil, in his third Georgic.

The modern *Hippomane* is the *manchineel tree*, a very acrid and deleterious plant, but the poisonous properties of which have been much exaggerated. Jacquin and Ricord have shewn that the notion formerly prevalent of the shade or *exhalations* from the tree being deleterious, is untrue, for they remained under its shadow for several hours, and even passed through extensive forests of manchineel trees without being injured. The sap which exudes when the boughs are cut or broken, is, however, very acrid; it will blister and sphacelate the skin, and, if inserted into wounds, cause death. The timber is beautifully variegated with brown and white, and capable of receiving a high polish; but before the trees are felled fires are usually lighted round them, to inspissate the sap, and render the hewer's occupation less dangerous; for, even when the juices are not applied in sufficient quantities to produce death, they cause intense pain, likened to the burning of a red-hot iron. This led to the cruel practice mentioned by Mérat and Lens, as having prevailed in the French colonies, of the slave-drivers steeping their scourges in manchineel juice before they flogged the negroes. When such enormities are perpetrated by the masters the sequel cannot excite surprise; for further on, we are told that the poisonous juice and fruit of the manchineel have been mixed by the slaves in coffee, to release themselves from their oppressors. M. Ricord states, from much experience, that the usual antidotes against this poison are ineffectual, and that the only remedy he found of use was an emulsion made from the seeds of the Nhandirhoba, (*feuillœ scandens*,) and, when the dose of *manchineel* had not been very large, this lessened and removed its effects.—*Burnett's Outlines of Botany.*

CASE OF ULCERATION OF THE EYELID.

Thomas Sprawson, ætat. fifty-nine, a builder, has an ulcer about as large as a sixpence at the inner canthus of the left eye.

*Characters of the Sore.* The surface of the ulceration is a little depressed and rather pale and smooth; its edges are slightly raised, indurated, and irregularly tuberculated; it discharges a thin watery fluid, which slightly irritates the surrounding skin; the sore is scarcely at all painful, but the contraction of the surrounding parts has drawn down the upper lid.

This patient is healthy in appearance, and says he has enjoyed a good state of health all his life, and is now pretty hearty.

*History.* A little pimple appeared at the inner canthus about three years ago; this was followed by two or three others, which arranged themselves in a somewhat circular manner, leaving a portion of healthy skin in the centre; this apparently healthy portion of skin ulcerated in the course of a few months, and obtained, as its margin, the tubercular elevation of the cutis, which I have just described. Since that period the ulceration has been extending, and has not appeared to be checked, influenced in the slightest degree by any measures his medical attendants have employed for his relief.

When I first saw this gentleman I placed him upon a regulated system of diet, and directed him to take, three times a day, four scruples of the carbonate of iron, and a few grains of blue pill and conium in the evening, and to apply to the sore a little black wash prepared with a large quantity of calomel. At this second visit the ulceration had completely healed, and his health had much improved, and this state of amendment still continues, from, as I believe, a rigid perseverance in the foregoing plan of treatment; and I am the more disposed to this opinion, from learning that his disease was increasing at the time I first saw him, and also from knowing that the same treatment has appeared to effect an equally desirable change in the condition of the other similar cases which, as I have stated, are now under my care.

After having given this account of the disease I have endeavoured to describe with as much accuracy as possible, it will be perceived that it only agrees with cancer in two circumstances, that is, in being preceded by induration, and in occurring after the middle period of life. It is easy to perceive in what respects it does not resemble cancer: it is much less painful and less rapid in its progress; it is more manageable by remedial agents, and is not infrequently cured by judiciously conducted treatment; and it does not stamp the countenance with those morbid appearances which are so peculiarly characteristic of a genuine malignant disease. It does not, in short, excite the same train and extent of constitutional symptoms which are present in all cancerous affections.—*Monthly Archives of the Medical Sciences, February.*

HOMŒOPATHY.

The Journal from which we are about to extract the following observations is a very curious one. It is called the "Monthly Journal of Medico-Chirurgical Knowledge, and is published at Paris in four languages, French, English, German, and Italian. The number now before us (for November, 1833,) is in English. It contains an anatomical plate, well engraved, and unusually distinct: the arteries and veins are coloured. It represents the "region occipito-claviculaire;" and "each number will contain a plate of topographical anatomy, drawn from nature, engraved on steel, and large as life." The English, which is very quaint and queer, is said to be the translation of Henry Belfield Lefevre. It is printed in double columns, and in a marvellously small type, rather calculated for those who love a good pennyworth, than for the comfort of elderly eyes. So much for externals. The articles are good; though we fear that the Anglo-Gallic phraseology in which they are couched will prevent many from perusing them. Among others, it contains a very ingenious essay on Homœopathy, by J. Boudet, who signs himself "Chemist and Druggist, Doctor of Sciences." Dr. Boudet does not believe in homœopathy as a whole, but only in one of its principles, viz. that an immense change is caused in the therapeutic effect of drugs by the manipulations to which they may be subjected. He observes that,

"Before Toricelli and Pascal, the pressure of the atmosphere was not entered as a necessary element in the formulæ of natural philosophy; and, before Lavoisier, the influence of its elements in all chemical combinations had scarcely been noticed; but it is to the labour of the last few years we owe the wondrous strides that analytical science has made: these labours it is that have revealed unto us, in chemical and physical agents, new forces, of which we did not even suspect the existence. In the following article I wish to shew the correlation existing between the subtile forces I just alluded to, and those which the German doctor pretends to have unveiled; and at the same time to indicate the many consequences which may result from this examination, both in therapeutics and pharmacy.

"Among the most remarkable effects of these forces we must class the phenomena of that voltaic electricity, which plays so grand a part in all the motions of matter, and which seems to extend its influence even over its slightest modifications. It is well known that the influence of that omnipresent force has been entirely substituted to the vague idea of chemical affinity; that the simple contact of any two bodies suffices to bring it into action; and that many liquids, for instance, are notably modified by the vases that contain them, although they enter not into combination with their substance. Thus, the infusion of violets preserves its colour much longer in a pewter vase than in any other; and the precise experiments of Dr. Bouchardat* have proved that the length of time during which milk can remain unaltered depends in a great measure on the vases in which it is contained: thus, in china, glass, or lead, it may coagulate in the course of three days, whilst in copper or brass it may remain liquid for a week. Fermentiscible liquids also undergo strange influences from the metal with which they come in contact. Dr. Bouchardat has demonstrated that alcoholic fermentation is almost immediately suspended by pouring the fermenting substances into vessels of brass or copper; and that alcohol placed in contact with mercury can no longer undergo acetic fermentation, whilst it absorbs oxygen, and becomes acid, as soon as it is removed from the influence of that metal.

" Again, the discovery of isomery† has signalized in inanimate matter a mobility of which we before had no conception, has unrolled before us a new world for our observation, and taught us to guard against apparent analogies which disappear before a more profound analysis. Thus, phosphoric acid, and its divers salts, which by chemists have been looked upon as identical as long as their analysis gave the same proportions of oxygen and phosporus, are now divided into phosphoric and pyro-phosphoric acids, phosphates and pyro-phosphates; and it has been fully proved, that the sole influence of heat might deeply modify certain mineral substances, although its action only extended to the position of their constituent molecules, without in the least altering their relative proportions.

" If such be the case with substances which their very constitution renders far less impressionable than organic matter, surely in organic matter similar phenomena must be far more common; and such is really the case. I shall confine myself to one example: it results from the late experiments of Mr. Robiquet,‡ that meconic acid, when heated in water to 100° (therm. cent.) is converted into parameconic acid; consequently, the extract of opium, prepared by evaporation on fire, may be modified as to its chemical and medicinal properties, by a variation of a few degrees on the temperature of the mixture."

* "Journal de Pharmacie, Sept. 1833."

† "*Isomérie*, a name given to a recently discovered effect produced on some bodies by certain influences which modify the constituent molecules of those bodies, so as to alter their relation of position, without altering their relation of proportion. Thus, to cite a familiar example, lump sugar may, by trituration, be partially converted into starch. Every one may have remarked, that when finely powdered sugar is dissolved in water, the liquid always remains turbid."—Trans.

‡ "Journal de Pharmacie, Sept. 1833; tome 18."

PISCIDIA ERYTHRINA, OR THE FISH-WOOD.

This is one of the plants which have the property of intoxicating fish. The following passages, however, (for which we are indebted to No. 19 of the "Outlines of Botany,") relate to its effects upon man. They are taken from a paper by Dr. Hamilton, read before the Medico-Botanical Society.

"My tincture was prepared by macerating one ounce of the coarsely powdered bark in twelve ounces, by measure, of rectified spirit, which I had brought with me from England, for twenty-four hours, and straining. The tincture thus obtained was of a fine honey yellow, and appeared to be fully impregnated with the active principle of the bark; it had nothing striking or offensive in its taste or smell, but, on being dropped into water, it communicated to it an opaline or milky hue, evidently from the separation of a resin; and, on suffering some of the undiluted tincture to evaporate in a glass, the sides were encrusted with a white film of the resin which remained behind. Labouring at the time under a severe toothach, which seemed to set sleep at defiance, I took towards night a drachm measure of this tincture in a tumbler of cold water, and lay down, with the uncorked phial in the one hand and the empty glass in the other, to speculate upon the manner of its operation on the system. The dose was by no means disagreeable to take, nor was its action on the mouth and throat so unpleasant as that of the bark in substance, which irritated the fauces like the Daphne Mezereum or the croton oil; but, soon after swallowing the dose, I became sensible of a burning sensation in the epigastric region, spreading rapidly to the surface, and terminating in a copious diaphoresis, in the midst of which I was surprised by a sleep so profound that I was utterly unconscious of existence from about eight o'clock at night till eight the following morning, when I awoke free from pain of every description, and found myself still grasping the uncorked phial in one hand, from which not a drop had been spilled, and the empty glass in the other. No unpleasant sensation followed, as is usually the case after opiates, from the exhibition of what was perhaps a needlessly large dose; nor did a friend, whom, though in perfect health, I persuaded to repeat my experiment in his own person, suffer the slightest inconvenience from an equally full dose: his only observation was, that he never had slept so sound in his life as he did that night. I next tried its efficacy as a topical application in cases of carious teeth, introducing a pledget of cotton impregnated with the tincture into the cavity, and never knew an instance of a return of pain after this application. I was next desirous of comparing its effects upon animalculæ in water with those of the tincture of opium: for this purpose I took, in two separate wine-glasses, equal quantities by measure of water, filled with the lively young of the mosquito, adding to the water in one glass a sufficient number of drops of the Tinctura opii to stupify the animalculæ, which fell in a mass to the bottom; I then dropped into the other an equal number of drops of the Tinctura piscidiæ, with a similar result. Next, taking the first glass, and carefully decanting the water without disturbing the insensible mass of animalculæ, I poured upon them fresh portions of pure water, previously filtered, in order to prevent confusion: upon which they revived, and swam about as actively as if nothing had happened. I treated those in the glass to which the dogwood tincture had been added, but without the slightest effect: the most frequently repeated affusions of pure water were not of the least avail; the animalculæ were truly dead, and thus furnished a conclusive proof of the superior potency of the dogwood over the opium tincture, in equal quantities."

### REDUCTION OF STRANGULATED HERNIA.

I would not say that nothing ought to be done before reduction is attempted, but this much I will venture to advise, that if the surgeon is called in very shortly after the strangulation has commenced, there ought to be no hesitation about commencing the reduction by the taxis, unless the patient should have a fever: in that case, a large portion of blood ought to be abstracted, and the cold bath might be used as Mr. G. directs. The method I use is this. I place the patient in a recumbent position, with the knees drawn up, and his body with the shoulders drawn towards the pelvis, so as to bring the intestines down towards the pubis; then I grasp the tumor with both hands, or, if it is very small, with my fingers; then, instead of shoving up the tumor towards the abdominal ring, I gently pull down the contents of the tumor, and, if possible, the included intestines; thus removing the obstruction at the ring, when, by gentle but steady compression, the air is forced out of the intestine, and the strangulation will be instantly relieved.

[The above is extracted from an article by Dr. Martin, in the *Baltimore Medical Journal*. He says that, by this method, for which he acknowledges himself indebted to Mr. Geohegan, of Dublin, he has succeeded in every instance but one.—Ed. *Med. Quart. Rev.*]

### FŒTUS OF THE WHALE.

M. Roussel de Vauzème presented the Academy of Sciences with a plaster model of the fœtus of a whale, extracted from its mother's womb, in the environs of the island of Tristan d'Acuntha (Atlantic Ocean). Peter Camper was yet the only naturalist who had had in his possession a whale fœtus: he has given a description of it in his posthumous works. The fœtus presented by Dr. Roussel weighs fifteen pounds, and measures two feet four inches in length. The time of gestation of the whale being from nine to twelve months, and the young whale being at its birth from twelve to fifteen feet long, the age of this fœtus may be approximately estimated at two months.—*Monthly Journal of Medico-Chirurgical Knowledge*.

## Figures and Tables

**Figure f1:**
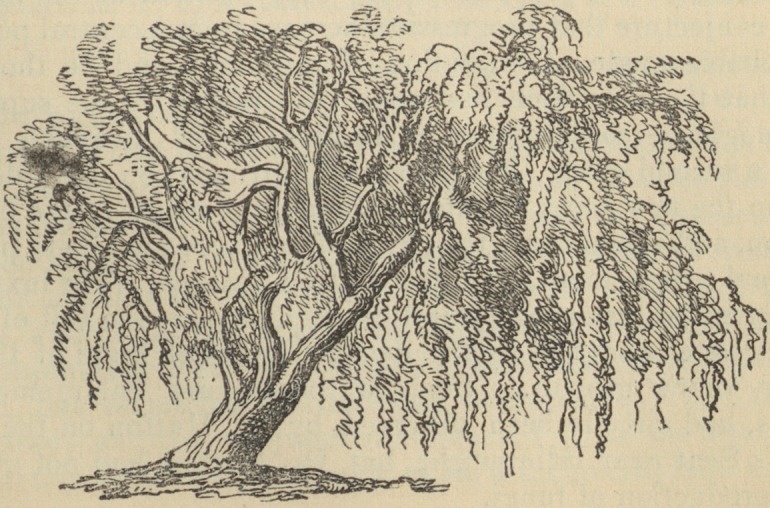


**Figure f2:**
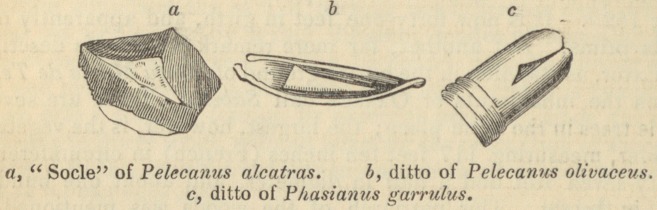


**Figure f3:**
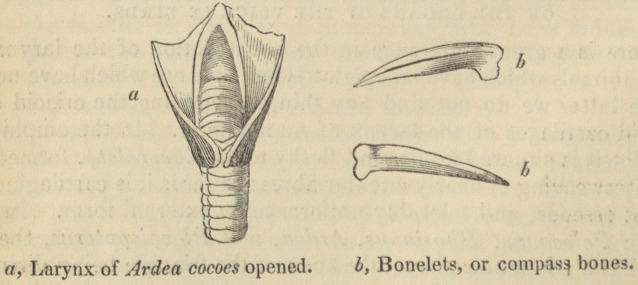


**Figure f4:**
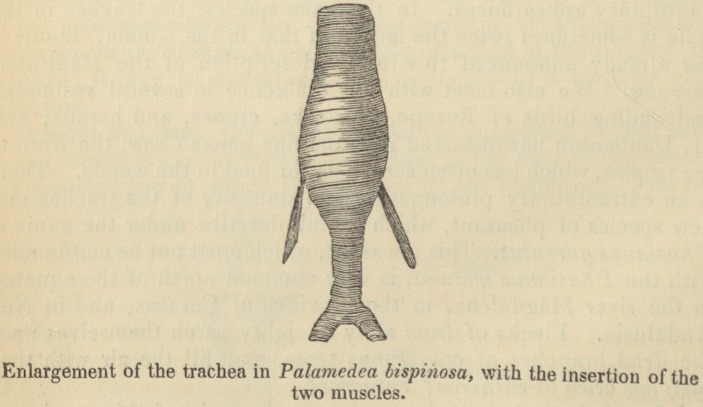


**Figure f5:**